# Comparison of brexpiprazole, aripiprazole, and placebo for Japanese major depressive disorder: A systematic review and network meta‐analysis

**DOI:** 10.1002/npr2.12414

**Published:** 2024-01-14

**Authors:** Taro Kishi, Kenji Sakuma, Takeo Saito, Atsuo Nakagawa, Masaki Kato, Nakao Iwata

**Affiliations:** ^1^ Department of Psychiatry Fujita Health University School of Medicine Toyoake Japan; ^2^ Department of Neuropsychiatry, School of Medicine St. Marianna University Kawasaki Japan; ^3^ Department of Neuropsychiatry Kansai Medical University Osaka Japan

**Keywords:** aripiprazole, brexpiprazole, Japanese major depressive disorder, network meta‐analysis, systematic review

## Abstract

**Aim:**

This systematic review and frequentist network meta‐analysis used random‐effects models is conducted to determine whether there are differences in the efficacy, acceptability, tolerability, and safety profiles of brexpiprazole (BRE) and aripiprazole (ARI) for Japanese with major depressive disorder (MDD) who were inadequately responsive to antidepressants.

**Methods:**

Outcome measures were scores on the Montgomery Åsberg Depression Rating Scale (primary), the Clinical Global Impression severity scale, and social functioning scale; the non‐response rate; the non‐remission rate; all‐cause discontinuation; discontinuation due to adverse events (DAE); at least one adverse event (1AE); serious adverse event, akathisia; tremor; weight gain.

**Results:**

A literature search identified three double‐blind, randomized, placebo‐controlled trials. These comprised one BRE study (with a 1 mg/day [BRE1] and a 2 mg/day [BRE2]) and two ARI studies (with a 3 mg/day arm and a flexible‐dose arm[within the dosage range approved in Japan]) (*n* = 1736). Both BRE and ARI demonstrated better efficacy than the placebo. BRE but not ARI had a higher DAE than the placebo. ARI but not BRE had a higher 1AE than the placebo. BRE and ARI had a higher risk of akathisia and weight gain than the placebo. There were no significant differences between BRE and ARI for any of the outcomes. Although BRE1 had good efficacy, it carried risk of weight gain. Although BRE2 also had efficacy, it carried risks of DAE, akathisia, and weight gain. However, the risk of akathisia in BRE2 was reduced by an initial dose of 0.5 mg/day rather than 1.0 mg/day.

**Conclusions:**

Overall BRE showed similar utility to ARI and a good risk–benefit balance.

## Abstract 2

This network meta‐analysis is conducted to determine whether there are differences in the efficacy, acceptability, tolerability and safety profiles of brexpiprazole and aripiprazole for Japanese with major depressive disorder. Overall, brexpiprazole showed similar utility to aripiprazole and a good risk‐benefit balance. The risk of akathisia in brexpiprazole 2 mg/day was reduced by an initial dose of 0.5 mg/day rather than 1.0 mg/day.

## Graphical abstract

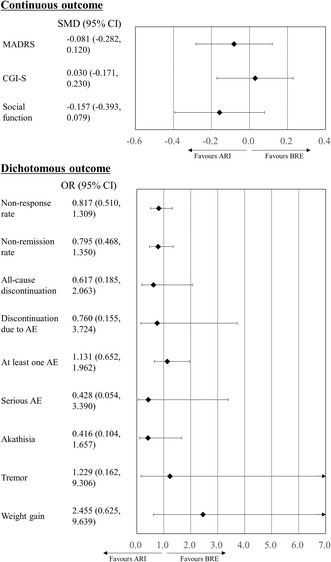

## 1 INTRODUCTION

Several treatment guidelines recommend the use of antipsychotics such as aripiprazole (ARI) for the treatment of antidepressant‐resistant major depressive disorder (AR‐MDD).^1^, ^2^, ^3^, ^4^, ^5^ Brexpiprazole (BRE) is an antipsychotic and, like ARI, it is a partial agonist of dopamine D2 and serotonin 5‐HT1A receptors and an antagonist of serotonin 5‐HT2A receptors.^6^, ^7^, ^8^ BRE has stronger dopamine D2‐blocking effects than ARI.^6^, ^7^ A Japanese phase 2/3 study has shown BRE at both 1 mg/day (BRE1) and 2 mg/day (BRE2) to be an effective, well‐tolerated adjunctive therapy for AR‐MDD.^9^ In the current study, we aimed to determine whether there were significant differences in the clinical value of BRE and ARI for Japanese patients with AR‐MDD. To compare the efficacy, acceptability, tolerability, and safety of the two drugs in this patient population, we conducted a systematic review and network meta‐analysis. This included only Japanese patients with AR‐MDD for the following reasons. First, Japan and other countries have different dose ranges for BRE in patients with AR‐MDD. The maximum dose in Japan is 2 mg,^9^; while it in the USA is 3 mg,^10^ respectively. Correlations have been found between the dose of antipsychotic drugs and the incidence of adverse events. For example, the risk of extrapyramidal side effects increases dose‐dependently.^11^ Second, empirical clinical studies suggest that East Asian patients may require lower dosages of psychotropic drugs, such as antipsychotics, lithium, and antidepressants, than non‐Asians.^12^ Racial and ethnic groups differ genetically in the activity of cytochrome P450, which participates in the metabolism of many drugs.^12^ Cytochrome P450 2D6 (CYP2D6) is involved in the metabolism of more than 20% of clinically prescribed drugs, including such as BRE and ARI.^13^ Because the CYP2D6 gene is highly polymorphic, individuals exhibit varying degrees of CYP2D6 enzyme activity, which could affect the safety and efficacy of drugs cleared and/or activated by CYP2D6.^14^ A recent pharmacokinetic study reported that the plasma concentrations of some antipsychotics, including BRE, metabolized by specific CYP enzymes, might be higher with the same daily dose in East Asian populations than in Western populations.^12^ A Japanese study has shown BRE1 to be effective in people with AR‐MDD^9^; however, an international study with predominantly Caucasian participants did not.^15^ The discrepancies between clinical studies in Japan and other countries are likely explained by the differences in drug metabolism activity between different ethnic groups.

## 2 METHODS

This study was conducted in accordance with the Preferred Reporting Items for Systematic Reviews and Meta‐Analyses guidelines^16^ (Appendix S1) and was registered on the Open Science Framework (https://osf.io/c38kn). The authors TK and KS double‐checked the literature search, data transfer accuracy, and calculations.

### 2.1 Search strategy and inclusion criteria

Detailed information about our literature search strategy is provided in Figure S1. We searched the PubMed, Cochrane Library, and Embase databases for studies published before November 16, 2023. The inclusion criteria for studies were (1) published and unpublished, double‐blind, randomized, placebo‐controlled trials of BRE or ARI as an adjunctive treatment for Japanese patients with AR‐MDD; (2) studies in which at least 70% of the participants were Japanese. The exclusion criteria were (1) open‐label studies; (2) studies in which there was a high risk of selection bias according to the Risk of Bias 2 tool^17^; (3) studies that included child/adolescent participants; (4) studies that included individuals with a dual diagnosis of MDD and another disorder such as substance use disorder; (5) studies that allowed the use of antipsychotics as a rescue medication during the trial; (6) studies that terminated early with no efficacy analysis.

### 2.2 Data synthesis, outcome measures, and data extraction

Outcome measures were Montgomery Åsberg Depression Rating Scale (MADRS)^18^ scores (the primary outcome for our study), Clinical Global Impression—severity (CGI‐S)^19^ scores, social function scale (Sheehan Disability Scale^20^ and Social Adaptation Self‐Evaluation Scale^21^) scores, the non‐response rate (response was defined as a reduction of ≥50% in MADRS total score from baseline at Week 6), the non‐remission rate (remission was defined as a response plus an absolute MADRS total score of ≤10), all‐cause discontinuation rate, discontinuation due to adverse events, at least one adverse event, serious adverse event, akathisia, tremor, and weight gain.

The extracted data were analyzed based on the intention‐to‐treat or full analysis set principles. If required data were missing from the studies, we searched for the data in published systematic review articles. We also attempted to contact the original investigators to obtain unpublished data.

### 2.3 Meta‐analysis methods

Both frequentist network and pairwise meta‐analyses were performed using a random‐effect model.^22^, ^23^ The primary network meta‐analysis compared the outcomes of BRE and ARI. For studies with two or more treatment arms of the same drug at different doses, data from the treatment arms were pooled for analysis so there were three treatment arms: BRE, ARI, and the placebo. In the secondary meta‐analysis, the drugs were divided by dose so there were five treatment arms: BRE1, BRE2, ARI 3 mg/day (ARI3), ARI flexible dose (ARI‐F), and the placebo. The final mean doses of the ARI‐F arm in the 2013 ARI study^24^ and the 2018 ARI study^25^ were 9.8 mg/day and 6.3 mg/day, respectively (the final mean dose of both ARI‐F arms was 8.0 mg/day). The standardized mean difference for continuous variables or the odds ratio for dichotomous variables was calculated with 95% confidence interval. Heterogeneity and inconsistency were evaluated.^26^, ^27^ In the secondary meta‐analysis, the surface under the curve cumulative ranking probabilities were used to rank the treatments for each outcome. The assumption of transitivity was evaluated by extracting potential effect modifiers (sample size, proportion of males, proportion of participants with recurrent episodes, and baseline MADRS score). The distributions of these were compared across the comparisons in the network. We classified the overall risk of bias for all trials using the Risk of Bias 2 tool.^17^ Finally, the results were incorporated into the Confidence in Network Meta‐Analysis (CINeMA) application, which is an adaptation of the Grading of Recommendations Assessment, Development, and Evaluation approach, to assess the credibility of the findings of each of the network meta‐analyses.^28^, ^29^, ^30^

We found BRE2 to be associated with a higher discontinuation due to adverse events and a higher incidence of akathisia than BRE1. There were correlations between the incidence of akathisia and both the current antipsychotic dose and the rate of dose increments.^31^ The initial dose in the Japan BRE for AR‐MDD study was 1 mg/day.^9^ However, our previous meta‐analysis^8^ found that the initial dose used in some international BRE studies with this patient population was 0.5 mg/day.^15^, ^32^, ^33^ Therefore, a subgroup pairwise meta‐analysis by initial dose (i.e., 0.5 mg/day or 1 mg/day) was also performed for the primary outcome, all‐cause discontinuation, discontinuation due to adverse events, and akathisia. Note that this subgroup pairwise meta‐analysis included studies with other ethnic groups as well as Japanese participants.

## 3 RESULTS

### 3.1 Study characteristics

A flowchart of our literature search strategy and a detailed explanation of the process are provided in Figure S1. In total, three double‐blind, randomized, placebo‐controlled trials (one BRE study^9^ and two ARI studies^24^, ^25^) were identified. In total, there were 1736 participants, 58.5% men, with a mean age of 39.5 years. The mean MADRS score at baseline was 26.0 and 53.0% of participants had experienced recurrent episodes of MDD. The characteristics of the studies included in the network meta‐analysis are summarized in Table 1. No clear evidence of violations was found on the transitivity assumption when we compared study characteristics across comparisons. We confirmed Otsuka Pharmaceutical Co., Ltd, the manufacturers of BRE that all domains in the Risk of Bias 2 tool for all studies included in our systematic review were evaluated as low risk. Consequently, all of the studies were evaluated as having a low overall risk of bias.

**TABLE 1:** Study characteristics.

Study name	Key inclusion criteria	AD trial prior to randomization	Treatment arm	*N*	AD	Mean age (mean ± SD)	Male	MADRS at baseline (mean ± SD)	Patients with recurrent episode
Kamijima 2013 (Japan)	MDD (DSM‐IV‐TR), HAMD17 ≥ 18, and inadequate response to 1–3 AD trials	Inadequate response (<50% reduction in the HAMD17), HAM‐D17 ≥ 14, or CGI‐I ≥ 3	ARI 3 mg/day	197	DUL (9.6%), FLU (20.0%), MIL (12.8%), PAR (19.3%), SER (38.4%)	38.7 ± 9.3	58.0%	25.3 ± 7.3	42.5%
			ARI 3–15 mg/day (final mean dose: 9.8 mg/day)	194					
			PLA	195					
Kamijima 2018^a^ (Japan and other countries)	MDD (DSM‐5), HAMD17 ≥ 18, and inadequate response to 1–3 AD trials	Inadequate response (<50% reduction in the HAMD17), HAM‐D17 ≥ 14, or CGI‐I ≥ 3	ARI 3–12 mg/day (final mean dose: 6.3 mg/d)	209	SER (100.0%)	38.9 ± 11.8	63.3%	25.0 ± 6.5	52.8%
			PLA	203					
Kato 2023 (Japan)	MDD (DSM‐5), HAMD17 ≥ 18, and inadequate response to 1–3 AD trials	Inadequate response (<50% reduction in the HAMD17), HAM‐D17 ≥ 14, or CGI‐I ≥ 3	BRE 1 mg/day	250	DUL (17.6%), ESC (30.7%), FLU (3.8%), MIL (2.4%), PAR (5.1%), SER (30.5%), VEN (9.3%)	40.2 ± 10.7	56.1%	27.0 ± 6.5	61.4%
			BRE 2 mg/day	245					
			PLA	243					

Abbreviations: AD, antidepressant; BRE, brexpiprazole; d, day; DSM, Diagnostic and Statistical Manual of Mental Disorders; DUL, duloxetine; ESC, escitalopram; FLUV, fluvoxamine; HAMD17, 17‐item Hamilton Depression Rating Scale of depression; MADRS, Montgomery Åsberg Depression Rating Scale; MDD, major depressive disorder; MIL, milnacipran; *N*, number of patients; PAR, paroxetine‐immediate release; PLA, placebo; SD, standard deviation; SER, sertraline; VEN‐XR, venlafaxine‐extended release.

^a^ Most participants were enrolled in Japan (72.7%).

### 3.2 Primary network meta‐analysis

**FIGURE 1:**
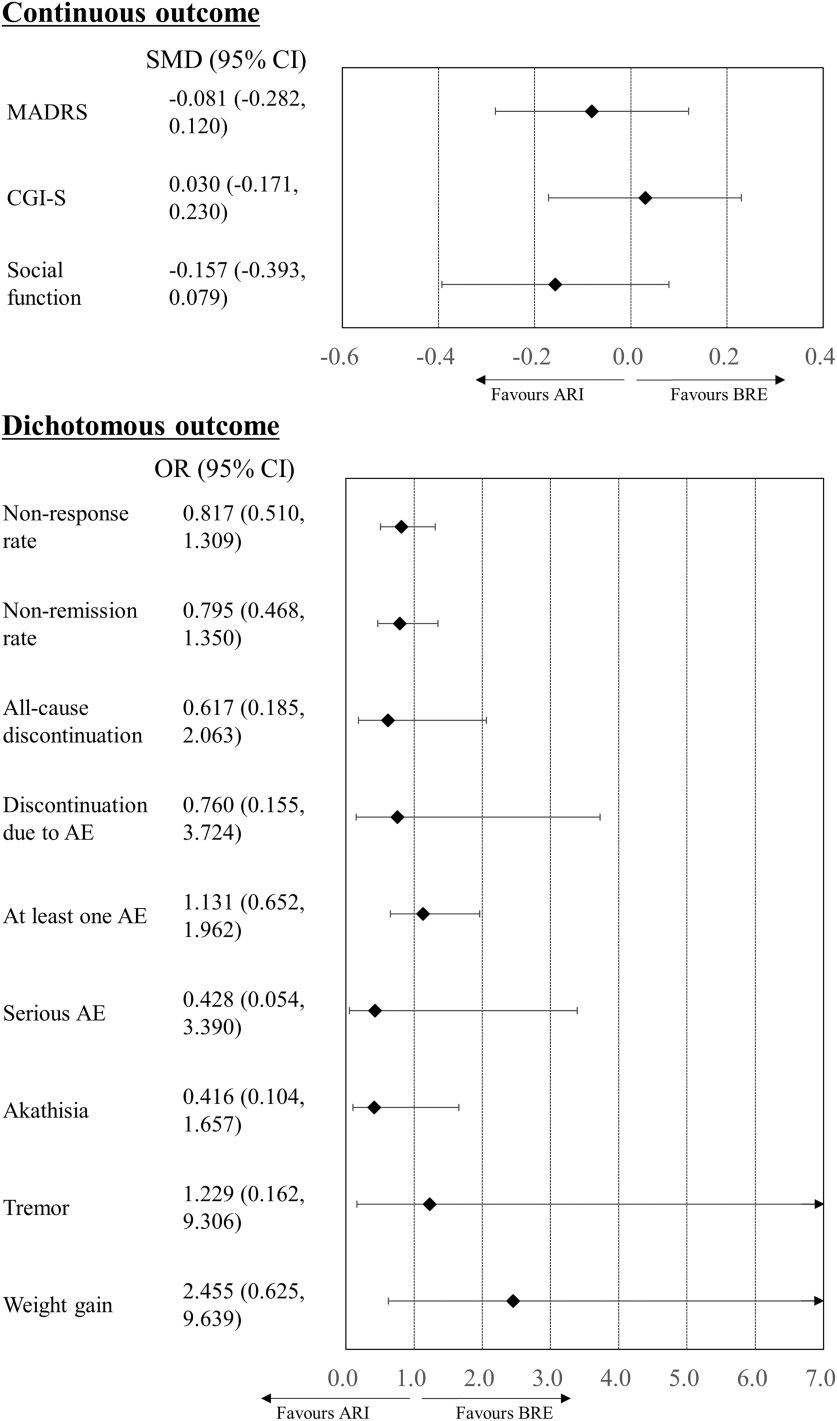
Results of a primary meta‐analysis. D2 partial agonists were compared with the placebo. Colors indicate the presence or absence of a significant difference: black, drug was similar to placebo. 95% CI, 95% confidence interval; AE, adverse event; ARI, aripiprazole; BRE, brexpiprazole; CGI‐S, Clinical Global Impression of illness Severity; MADRS, Montgomery Åsberg Depression Rating Scale; OR, odds ratio; PLA, placebo; SMD, standardized mean difference.

We showed a network plot in Figure S2. Both BRE and ARI were superior to the placebo in their improvement of MADRS scores, CGI‐S scores, and social function scale scores (Table 2). ARI but not BRE had the lower non‐response and non‐remission rates than the placebo (Table 2). Although BRE but not ARI had a higher rate of discontinuation due to adverse events than the placebo, ARI but not BRE had a higher incidence of at least one adverse event compared with the placebo (Table 2). BRE and ARI had higher risk of both akathisia and weight gain compared to the placebo (Table 2). There were no significant differences in any of the other outcomes between the antipsychotic groups and the placebo group (Table 2). There were also no significant differences between BRE and ARI for any of the outcomes (Figure 1 and Table 2). As the number of studies and patients included in this network meta‐analysis was small, we did not analyze global inconsistency, local inconsistency, or publication bias (Table S1). Local heterogeneity was evaluated for ARI only (Table S1). Consequently, confidence in the evidence in the primary network meta‐analysis was evaluated as low or very low.

**TABLE 2:** Results of a primary meta‐analysis.

MADRS (SMD [95% CI])		
ARI	−0.081 (−0.282, 0.120)	**−0.305 (−0.434, −0.176)**
	BRE	**−0.224 (−0.378, −0.070)**
		PLA
Non‐response rate (OR [95% CI])		
ARI	0.817 (0.510, 1.309)	**0.574 (0.434, 0.758)**
	BRE	0.702 (0.480, 1.027)
		PLA
Non‐remission rate (OR [95% CI])		
ARI	0.795 (0.468, 1.350)	**0.583 (0.430, 0.790)**
	BRE	0.733 (0.475, 1.132)
		PLA
CGI‐S (SMD [95% CI])		
ARI	0.030 (−0.171, 0.230)	**−0.162 (−0.290, −0.033)**
	BRE	**−0.191 (−0.345, −0.037)**
		PLA
Social function (SMD [95% CI])		
ARI	−0.157 (−0.393, 0.079)	**−0.427 (−0.575, −0.279)**
	BRE	**−0.270 (−0.454, −0.086)**
		PLA
All‐cause discontinuation (OR [95% CI])		
ARI	0.617 (0.185, 2.063)	1.018 (0.503, 2.060)
	BRE	1.650 (0.619, 4.396)
		PLA
Discontinuation because of adverse events (OR [95% CI])		
ARI	0.760 (0.155, 3.724)	2.700 (0.975, 7.478)
	BRE	**3.552 (1.049, 12.025)**
		PLA
At least one adverse event (OR [95% CI])		
ARI	1.131 (0.652, 1.962)	**1.665 (1.186, 2.339)**
	BRE	1.472 (0.954, 2.271)
		PLA
Serious adverse event (OR [95% CI])		
ARI	0.428 (0.054, 3.390)	0.634 (0.172, 2.334)
	BRE	1.482 (0.297, 7.395)
		PLA
Akathisia (OR [95% CI])		
ARI	0.416 (0.104, 1.657)	**5.948 (3.171, 11.158)**
	BRE	**14.311 (4.177, 49.024)**
		PLA
Tremor (OR [95% CI])		
ARI	1.229 (0.162, 9.306)	1.920 (0.578, 6.371)
	BRE	1.562 (0.306, 7.980)
		PLA
Weight gain (OR [95% CI])		
ARI	2.455 (0.625, 9.639)	**7.850 (2.748, 22.426)**
	BRE	**3.198 (1.331, 7.684)**
		PLA

*Note*: Drugs are reported alphabetically. Data are presented as odds ratios or standardized mean differences (95% confidence intervals) in the column‐defining treatment compared with the row‐defining treatment. Odds ratios <1 favor the row‐defining treatment. Standardized mean differences <0 favor the row‐defining treatment.

The boldface result indicates statistical significance.

Abbreviations: 95% CI, 95% confidence interval; ARI, aripiprazole; BRE, brexpiprazole; CGI‐S, Clinical Global Impression of illness Severity; MADRS, Montgomery Åsberg Depression Rating Scale; OR, odds ratio; PLA, placebo; SMD, standardized mean difference.

### 3.3 Secondary network meta‐analysis

**FIGURE 2:**
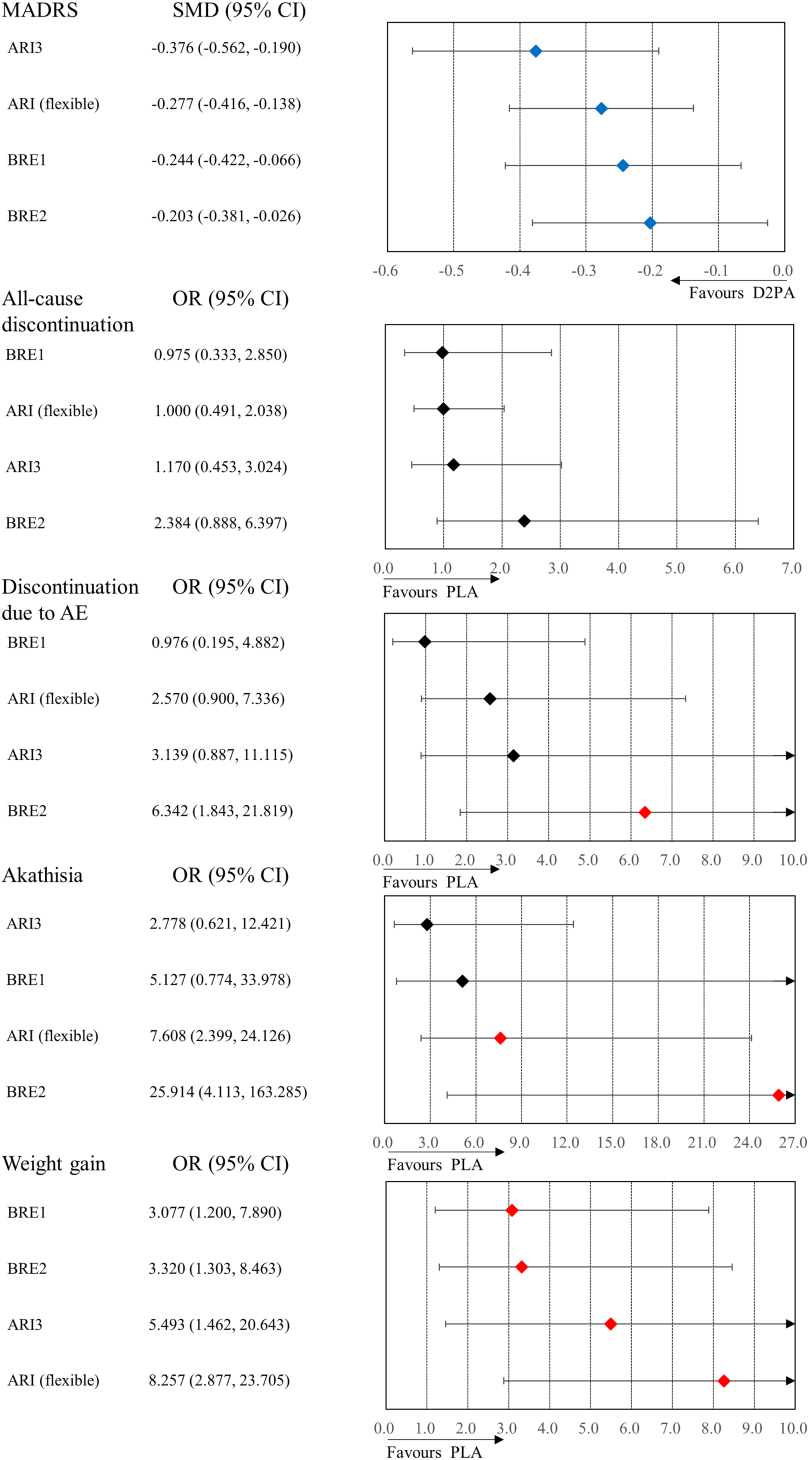
Results of a secondary meta‐analysis. D2 partial agonists were compared with the placebo. Colors indicate the presence or absence of a significant difference: blue, drug was superior to placebo; black, drug was similar to placebo; red, drug was inferior to placebo. The order of treatments is according to the mean effect size. 95% CI, 95% confidence interval; AE, adverse event; ARI, aripiprazole; BRE, brexpiprazole; D2PA, dopamine D2 partial agonist; MADRS, Montgomery Åsberg Depression Rating Scale; OR, odds ratio; PLA, placebo; SMD, standardized mean difference.

All active‐treatment arms (BRE1, BRE2, ARI3, and ARI‐F) outperformed the placebo in the improvement of MADRS scores, CGI‐S scores, and social function scale scores (Figure 2 and Table 3). ARI3 and ARI‐F, but not BRE1 and BRE2, also had a lower non‐response and non‐remission rates compared with the placebo (Table 3). BRE2 was associated with a higher rate of discontinuation because of adverse events than the placebo and BRE1 (Figure 2 and Table 3). ARI‐F was associated with a higher incidence of at least one adverse event than the placebo (Table 3). BRE2 and ARI‐F were associated with higher incidences of akathisia than the placebo (Figure 2). BRE1, BRE2, ARI3, and ARI‐F were associated with higher incidences of weight gain than the placebo (Figure 2). There were no significant differences in other outcomes between the antipsychotic groups and the placebo (Table 3). There were no significant differences in any of the outcomes between antipsychotic groups other than discontinuation due to adverse events (Table 3). As the number of studies and patients included in our network meta‐analysis was small, we did not analyze local inconsistency or publication bias (Table S2). We evaluated local heterogeneity for ARI‐F only (Table S2). Consequently, confidence in the evidence in the secondary network meta‐analysis was also evaluated as low or very low.

**TABLE 3:** Results of a secondary meta‐analysis.

MADRS (SMD [95% CI])				
ARI3	−0.099 (−0.285, 0.086)	−0.132 (−0.389, 0.125)	−0.173 (−0.430, 0.085)	**−0.376 (−0.562, −0.190)**
	ARI (flexible)	−0.033 (−0.258, 0.193)	−0.074 (−0.299, 0.152)	**−0.277 (−0.416, −0.138)**
		BRE1	−0.041 (−0.217, 0.136)	**−0.244 (−0.422, −0.066)**
			BRE2	**−0.203 (−0.381, −0.026)**
				PLA
Nonresponse rate (OR [95% CI])				
ARI3	0.897 (0.614, 1.311)	0.774 (0.434, 1.382)	0.737 (0.412, 1.319)	**0.531 (0.360, 0.783)**
	ARI (flexible)	0.863 (0.511, 1.457)	0.822 (0.485, 1.391)	**0.592 (0.439, 0.798)**
		BRE1	0.952 (0.633, 1.432)	0.686 (0.446, 1.054)
			BRE2	0.720 (0.467, 1.110)
				PLA
Nonremission rate (OR [95% CI])				
ARI3	0.902 (0.603, 1.348)	0.743 (0.390, 1.416)	0.733 (0.384, 1.400)	**0.541 (0.356, 0.823)**
	ARI (flexible)	0.824 (0.458, 1.484)	0.813 (0.451, 1.467)	**0.600 (0.434, 0.830)**
		BRE1	0.987 (0.621, 1.568)	0.729 (0.446, 1.190)
			BRE2	0.738 (0.451, 1.209)
				PLA
CGI‐S (SMD [95% CI])				
ARI3	−0.072 (−0.257, 0.113)	−0.086 (−0.342, 0.170)	0.042 (−0.215, 0.299)	**−0.213 (−0.399, −0.028)**
	ARI (flexible)	−0.014 (−0.238, 0.212)	0.114 (−0.112, 0.340)	**−0.141 (−0.280, −0.002)**
		BRE1	0.128 (−0.049, 0.304)	**−0.128 (−0.305, 0.050)**
			BRE2	**−0.255 (−0.433, −0.077)**
				PLA
Social function (SMD [95% CI])				
ARI3	0.039 (−0.160, 0.237)	−0.126 (−0.404, 0.151)	−0.132 (−0.410, 0.146)	**−0.396 (−0.596, −0.197)**
	ARI (flexible)	−0.165 (−0.410, 0.080)	−0.171 (−0.416, 0.075)	**−0.435 (−0.586, −0.285)**
		BRE1	−0.006 (−0.198, 0.187)	**−0.270 (−0.464, −0.076)**
			BRE2	**−0.264 (−0.459, −0.070)**
				PLA
All‐cause discontinuation (OR [95% CI])				
ARI3	1.170 (0.464, 2.953)	1.201 (0.287, 5.030)	0.491 (0.125, 1.932)	1.170 (0.453, 3.024)
	ARI (flexible)	1.026 (0.283, 3.718)	0.419 (0.124, 1.417)	1.000 (0.491, 2.038)
		BRE1	0.409 (0.152, 1.097)	0.975 (0.333, 2.850)
			BRE2	2.384 (0.888, 6.397)
				PLA
Discontinuation because of adverse events (OR [95% CI])				
ARI3	1.222 (0.450, 3.316)	3.217 (0.415, 24.923)	0.495 (0.085, 2.900)	3.139 (0.887, 11.115)
	ARI (flexible)	2.634 (0.386, 17.997)	0.405 (0.080, 2.049)	2.570 (0.900, 7.336)
		BRE1	**0.154 (0.045, 0.529)**	0.976 (0.195, 4.882)
			BRE2	**6.342 (1.843, 21.819)**
				PLA
At least one adverse event (OR [95% CI])				
ARI3	0.828 (0.415, 1.653)	1.304 (0.495, 3.437)	0.748 (0.282, 1.988)	1.478 (0.749, 2.916)
	ARI (flexible)	1.575 (0.667, 3.718)	0.904 (0.379, 2.153)	**1.784 (1.071, 2.973)**
		BRE1	0.574 (0.284, 1.158)	1.133 (0.568, 2.261)
			BRE2	1.975 (0.979, 3.985)
				PLA
Akathisia (OR [95% CI])				
ARI3	0.365 (0.088, 1.518)	0.542 (0.049, 6.045)	0.107 (0.010, 1.150)	2.778 (0.621, 12.421)
	ARI (flexible)	1.484 (0.162, 13.598)	0.294 (0.033, 2.578)	**7.608 (2.399, 24.126)**
		BRE1	**0.198 (0.043, 0.921)**	5.127 (0.774, 33.978)
			BRE2	**25.914 (4.113, 163.285)**
				PLA
Tremor (OR [95% CI])				
ARI3	0.911 (0.156, 5.314)	1.070 (0.075, 15.269)	1.427 (0.099, 20.666)	1.911 (0.303, 12.032)
	ARI (flexible)	1.175 (0.110, 12.505)	1.566 (0.145, 16.956)	2.097 (0.526, 8.368)
		BRE1	1.333 (0.201, 8.825)	1.785 (0.262, 12.153)
			BRE2	1.339 (0.193, 9.306)
				PLA
Weight gain (OR [95% CI])				
ARI3	0.665 (0.269, 1.646)	1.785 (0.352, 9.060)	1.655 (0.327, 8.370)	**5.493 (1.462, 20.643)**
	ARI (flexible)	2.683 (0.653, 11.030)	2.487 (0.607, 10.185)	**8.257 (2.877, 23.705)**
		BRE1	0.927 (0.474, 1.812)	**3.077 (1.200, 7.890)**
			BRE2	**3.320 (1.303, 8.463)**
				PLA

*Note*: Drugs are reported alphabetically. Data are presented as odds ratios or standardized mean differences (95% confidence intervals) in the column‐defining treatment compared with the row‐defining treatment. Odds ratios <1 favor the row‐defining treatment. Standardized mean differences <0 favor the row‐defining treatment.

The boldface result indicates statistical significance.

Abbreviations: 95% CI, 95% confidence interval; ARI, aripiprazole; BRE, brexpiprazole; CGI‐S, Clinical Global Impression of illness Severity; MADRS, Montgomery Åsberg Depression Rating Scale; OR, odds ratio; PLA, placebo; SMD, standardized mean difference.

### 3.4 Subgroup meta‐analysis divided by different BRE initial doses

The characteristics of the four studies included in this meta‐analysis are summarized in Table S3.^9^, ^15^, ^32^, ^33^ While an initial BRE dose of 1 mg/day was associated with a larger akathisia effect size than an initial dose of 0.5 mg/day, none of the other outcomes differed significantly in the magnitude of the effect size between an initial dose of 1 mg/day and an initial dose of 0.5 mg/day (Table 4).

**TABLE 4:** Subgroup pairwise meta‐analysis of studies of brexpiprazole use with antidepressant‐resistant major depressive disorder divided according to titration methods. (A). Brexpiprazole 2 mg/day versus placebo. (B). Brexpiprazole 1 mg/day versus placebo.

(A).	Titration method	*K*	*n*	OR (95% CI)	*p*	*I* ^2^	Test for subgroup difference
All‐cause discontinuation	1st week: 0.5 mg/day 2nd week: 1.0 mg/day 3rd week: 2.0 mg/day	2	773	1.67 (0.68, 4.10)	0.26	53.0%	*p* = 0.54, *I* ^2^ = 0.0%
	1st week: 1 mg/day 2nd week: 2.0 mg/day	1	490	2.38 (1.18, 4.82)	0.02	NA	
Discontinuation due to adverse event	1st week: 0.5 mg/day 2nd week: 1.0 mg/day 3rd week: 2.0 mg/day	2	773	6.55 (1.14, 37.68)	0.04	0.0%	*p* = 0.98, *I* ^2^ = 0.0%
	1st week: 1 mg/day 2nd week: 2.0 mg/day	1	490	6.34 (1.84, 21.82)	0.003	NA	
Akathisia	1st week: 0.5 mg/day 2nd week: 1.0 mg/day 3rd week: 2.0 mg/day	2	773	3.19 (0.75, 13.47)	0.12	66.0%	*p* = 0.03, *I* ^2^ = 79.5%
	1st week: 1 mg/day 2nd week: 2.0 mg/day	1	490	25.91 (8.00, 83.93)	<0.00001	NA	
	Titration method	*K*	*n*	SMD (95% CI)	*p*	*I* ^2^	Test for subgroup difference
MADRS	1st week: 0.5 mg/day 2nd week: 1.0 mg/day 3rd week: 2.0 mg/day	2	771	−0.33 (−0.47, −0.19)	<0.00001	0.0%	*p* = 0.29, *I* ^2^ = 11.9%
	1st week: 1 mg/day 2nd week: 2.0 mg/day	1	488	−0.20 (−0.38, −0.03)	0.02	NA	

(B).	Titration method	*K*	*n*	OR (95% CI)	*p*	*I* ^2^ (%)	Test for subgroup difference
All‐cause discontinuation	1st week: 0.5 mg/day 2nd week: 1.0 mg/day	1	447	0.74 (0.32, 1.73)	0.49	NA	*p* = 0.65, *I* ^2^ = 0.0%
	No titration	1	494	0.97 (0.43, 2.21)	0.95	NA	
Discontinuation due to adverse event	1st week: 0.5 mg/day 2nd week: 1.0 mg/day	1	447	0.98 (0.20, 4.90)	0.98	NA	*p* = 1.00, *I* ^2^ = 0.0%
	No titration	1	494	0.98 (0.20, 4.88)	0.98	NA	
Akathisia	1st week: 0.5 mg/day 2nd week: 1.0 mg/day	1	447	2.00 (0.67, 5.95)	0.21	NA	*p* = 0.27, *I* ^2^ = 19.0%
	No titration	1	494	5.13 (1.47, 17.94)	0.01	NA	
	Titration method	*K*	*n*	SMD (95% CI)	*p*	*I* ^2^	Test for subgroup difference
MADRS	1st week: 0.5 mg/day 2nd week: 1.0 mg/day	1	443	−0.17 (−0.36, 0.01)	0.07	NA	*p* = 0.59, *I* ^2^ = 0.0%
	No titration	1	491	−0.24 (−0.42, −0.07)	0.007	NA	

Abbreviations: 95% CI, 95% confidence interval; d, day; *K*, number of studies; MADRS, Montgomery Åsberg Depression Rating Scale; *n*, number of participants; NA, not applicable; OR, odds ratio; SMD, standardized mean difference.

## 4 DISCUSSION

BRE and ARI were efficacious treatments for Japanese patients with AR‐MDD, and both could be considered more acceptable to those patients based on the lack of significant differences in all‐cause discontinuation between the two antipsychotics and the placebo. However, tolerance was a concern with BRE as it carried a risk of discontinuation due to adverse events. Safety was a concern with ARI as it was associated with a higher incidence of at least one adverse event compared with the placebo. Both BRE and ARI carried risks of akathisia and weight gain. Thus, clinicians should perform physical and mental examinations before prescribing these antipsychotics for Japanese patients with AR‐MDD.

It may be possible to reduce the tolerance and safety concerns. BRE1 had similar efficacy to BRE2 but was associated with a lower discontinuation due to adverse events. Moreover, BRE1 did not increase the risk of akathisia. Similarly, ARI3 had similar efficacy to ARI‐F (mean dose, 8.0 mg/day), there were no significant differences in the incidence of at least one adverse event and akathisia between ARI3 and the placebo. Therefore, clinicians should exercise caution when increasing the dosage of these antipsychotics to avoid adverse events.

The initial dose of BRE may be associated with the incidence of adverse events. Even when the dose was increased to BRE2 over several weeks, akathisia appeared less frequently when the initial dose was 0.5 mg/day than when it was 1 mg/day. Although there were no significant correlations found between the magnitude of odds ratio and differences in initial dose for other outcomes, an initial dose of 0.5 mg/day might improve acceptability, tolerance, and safety for those with AR‐MDD than an initial dose of 1 mg/day. Because the results might be associated with variation in BRE metabolism among the population, a study with an initial dose of 0.5 mg/day BRE for Japanese patients with AR‐MDD should be conducted.

ARI but not BRE produced more responses and remissions than a placebo. The Defined Daily Dose (DDD) for ARI3 was approximately 0.2. The dose of BRE that corresponds to a DDD of 0.2 is 0.6 mg/day. A recent pharmacokinetic study reported that the plasma concentrations of antipsychotics metabolized by specific CYP enzymes, including BRE, may be higher in East Asian populations than in Western populations at the same daily dose.^12^ Thus, because BRE 0.5 mg/day may have better efficacy, acceptability, tolerability, and safety for Japanese patients with AR‐MDD, a double‐blind, randomized, placebo‐controlled trial for Japanese patients with AR‐MDD that includes a BRE 0.5 mg/day arm should be conducted.

Our study had several limitations. First, because the number of studies and participants was small, we could not sufficiently evaluate the heterogeneity and inconsistency. Moreover, because there was not a direct comparison of BRE with ARI for AR‐MDD, the results of the network meta‐analysis comparing BRE with ARI for all outcomes were only indirect evidence. Therefore, we determined that there was low or very low confidence in the evidence for most of the outcome comparisons. Second, we did not examine whether blood concentrations of the two antipsychotics were associated with efficacy, acceptability, tolerability, and safety outcomes. Third, we did not examine which antidepressants were compatible with each antipsychotic.

In conclusion, BRE showed similar utility to ARI for Japanese patients with AR‐MDD. BRE1 showed a good risk–benefit balance for Japanese patients with AR‐MDD although BRE1 had a risk of weight gain. BRE2 was efficacious but carried risks of discontinuation due to adverse events, akathisia, and weight gain. However, the risk of akathisia may be reduced by an initial dose of 0.5 mg/day rather than 1.0 mg/day.

## AUTHOR CONTRIBUTIONS

TK developed the study concept and design and performed the statistical analyses. TK and SK took full responsibility for the data integrity and the accuracy of the data analysis. All authors interpreted the data and wrote the manuscript. NI supervised the review.

## FUNDING INFORMATION

Grant‐in‐Aid for Scientific Research (C) (23K06998).

## CONFLICT OF INTEREST STATEMENT

The authors have no specific conflicts of interest to declare concerning this study. They would like to disclose the following interests that have arisen in the last 3 years: TK has received speaker's honoraria from Eisai, Janssen, Meiji, MSD, Otsuka, Sumitomo, Takeda, Tanabe‐Mitsubishi, and Viatris and research grants from Eisai, Grant‐in‐Aid for Scientific Research© (19 K08082 and 23 K06998), Japan Agency for Medical Research and Development (JP22dk0307107, JP22wm0525024, JP23dk0307117, JP23wm0525024, and JP23dk0307122), and the Japanese Ministry of Health, Labour and Welfare (21GC1018). KS has received speaker's honoraria from Eisai, Janssen, Kyowa, Meiji, Otsuka, Sumitomo, and Takeda and research grants from Grant‐in‐Aid for Young Scientists (19 K17099), Grant‐in‐Aid for Scientific Research© (23 K06998), Fujita Health University School of Medicine Research Grant for Early‐Career Scientists, and Japan Agency for Medical Research and Development (JP22dk0307107). TS has received speaker's honoraria from Sumitomo and research grants from Grant‐in‐Aid for Scientific Research© (21 K07490) and Japan Agency for Medical Research and Development (JP22dk0307115). AN received speaker's honoraria from Lundbeck, Takeda, Otsuka, Sumitomo, Viatris, and Yoshitomi yakuhin, and consulting fees from Otsuka and MICIN and research grants from Grant‐in‐Aid for Scientific Research (20H01772) and Japan Agency for Medical Research and Development (JP23dk0307116). MK received consulting fees from Sumitomo, Otsuka, Lundbeck, Takeda, and Shionogi; and payment/honoraria from Sumitomo, Otsuka, Meiji, Eli Lilly, MSD, Pfizer, Janssen, Shionogi, Mitsubishi Tanabe, Takeda, Lundbeck, Viatris, Eisai, Kyowa, and Ono and has received grant funding from the Japan Society for the Promotion of Science (22K07607), Japan Agency for Medical Research and Development (JP20dk0307081), SENSHIN Medical Research Foundation, the Japan Research Foundation for Clinical Pharmacology and the Japanese Society of Clinical Neuropsychopharmacology. NI has received speaker's honoraria from Eisai, Janssen, Meiji, Otsuka, Sumitomo, Takeda, Tanabe‐Mitsubishi, and Viatris and research grants from Daiichi Sankyo, Eisai, Meiji, Otsuka, Sumitomo, Takeda, and Tanabe‐Mitsubishi.

## ETHICAL APPROVAL

Approval of the research protocol by an Institutional Reviewer Board: N/A.

Informed Consent: N/A.

Registry and the Registration No. of the study/trial: N/A.

Animal Studies: N/A.

## Supporting information


Appendix S1

## Data Availability

All descriptive variables are openly available in the articles of the studies that are cited in this paper.
