# Supplementary material for: Comparison of brexpiprazole, aripiprazole, and placebo for Japanese major depressive disorder: A systematic review and network meta‐analysis
Source: Neuropsychopharmacol Rep. 2024 Jan 14;44(1):165–75. doi: 10.1002/npr2.12414 (PMC10932760; doi:10.1002/npr2.12414)
Supplement: Supplementary file 1 — Appendix S1 [file NPR2-44-165-s001.pdf]

Figure S1. Preferred Reporting Items for Systematic reviews and Meta-Analyses flow diagram

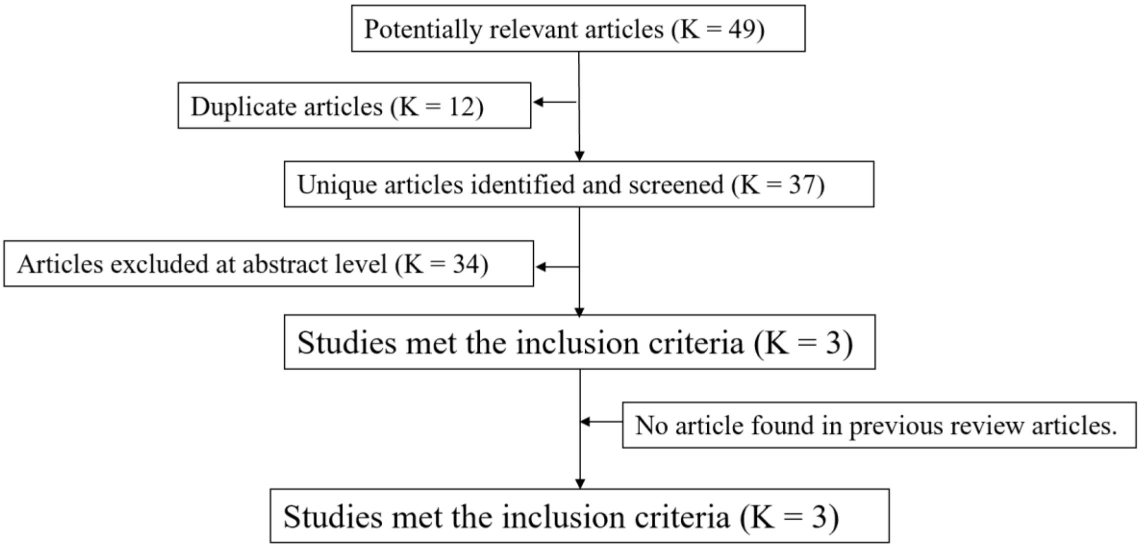

We searched the PubMed, Cochrane Library, and Embase databases for studies published before November 16, 2023. The search terms for PubMed, and the Cochrane Library included (aripiprazole OR brexpiprazole) AND (placebo) AND (random*) and (Japan*) and (major depressive disorder OR major depression). The search terms for Embase included ('major depression'/exp OR 'major depression') AND ('placebo'/exp OR placebo) AND ('aripiprazole'/exp OR aripiprazole OR 'brexpiprazole'/exp OR brexpiprazole) AND ('japan'/exp OR japan). The search terms for ICHUSHI (Japanese) included (aripiprazole OR brexpiprazole) AND (randomized OR double-blind). Additionally, reference lists of the included articles were manually searched for additional relevant published and unpublished research, including conference abstracts. We also searched clinical trial registries (ClinicalTrials.gov [http://clinicaltrials.gov/] and the World Health Organization International Clinical Trials Registry Platform [http://www.who.int/ictrp/search/en/]) to ensure the RCTs were comprehensive and to minimize the effect of publication bias. Moreover, we also used the drug package insert for each antipsychotic to determine search criteria.[1] Any discrepancies in the selected articles were resolved by consensus of the authors. If multiple papers or academic conference abstracts were reported despite the same research, the literature was screened by confirming the clinical trial registration number and/or reference to past review articles.

1. PMDA. Pharmaceuticals and Medical Devices Agency. https://www.pmda.go.jp/.

Review articles used in the hand search

1. Furukawa Y, Oguro S, Obata S, Hamza T, Ostinelli EG, Kasai K. Optimal dose of brexpiprazole for augmentation therapy of antidepressant-refractory depression: A systematic review and dose-effect meta-analysis. Psychiatry Clin Neurosci. 2022 Sep;76(9):416-422.

2. Hobart M, Zhang P, Weiss C, Meehan SR, Eriksson H. Adjunctive Brexpiprazole and Functioning in Major Depressive Disorder: A Pooled Analysis of Six Randomized Studies Using the Sheehan Disability Scale. Int J Neuropsychopharmacol 2019; 22: 173-179.

3. Katzman MA, Therrien F, MacKenzie EM, Wang F, de Jong-Laird A, Boucher M. Efficacy of adjunctive brexpiprazole on symptom clusters of major depressive disorder: A post hoc analysis of four clinical studies. J Affect Disord 2022; 316: 201-208.

4. Kishimoto T, Hagi K, Kurokawa S, Kane JM, Correll CU. Efficacy and safety/tolerability of antipsychotics in the treatment of adult patients with major depressive disorder: a systematic review and meta-analysis. Psychol Med 2023; 53: 4064-4082.

5. Newcomer JW, Meehan SR, Chen D, Brubaker M, Weiss C. Changes in Metabolic Parameters and Body Weight in Patients With Prediabetes Treated With Adjunctive Brexpiprazole for Major Depressive Disorder: Pooled Analysis of Short- and Long-Term Clinical Studies. J Clin Psychiatry 2023; 84.

6. Nunez NA, Joseph B, Pahwa M et al. Augmentation strategies for treatment resistant major depression: A systematic review and network meta-analysis. J Affect Disord 2022; 302: 385-400.

7. Wang J, Li W, Li M, Wu H, Qiu Z. Comparative efficacy and safety of 4 atypical antipsychotics augmentation treatment for major depressive disorder in adults: A systematic review and network meta-analysis. Medicine (Baltimore) 2023; 102: e34670.

8. Kishi T, Sakuma K, Nomura I, Matsuda Y, Mishima K, Iwata N. Brexpiprazole as Adjunctive Treatment for Major Depressive Disorder Following Treatment Failure With at Least One Antidepressant in the Current Episode: a Systematic Review and Meta-Analysis. Int J Neuropsychopharmacol 2019; 22: 698-709.

Figure S2. Network plot

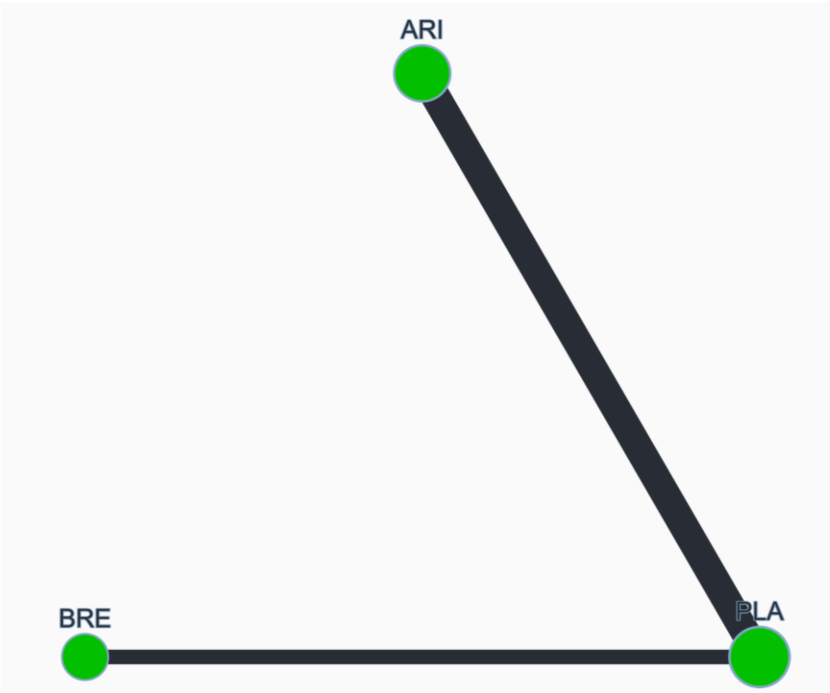

Node size by sample size

Node color by risk of bias

Green: low overall risk of bias

Edge width by number of studies

Table S1. Global and local heterogeneity and global inconsistency in the primary meta-analyses

	tau^{2*}	Design-by-treatment interaction model
MADRS	0.000	na
Non-response rate	0.000	na
Non-remission rate	0.000	na
CGI-S	0.000	na
Social function	0.003	na
All-cause discontinuation**	0.135	na
Discontinuation due to adverse events	0.000	na
At least one adverse event	0.023	na
Serious adverse event	0.000	na
Akathisia	0.042	na
Tremor**	0.539	na
Weight gain	0.000	na

*As previously suggested (Huhn 2019), the common tau^2 was compared to the empirical distributions of heterogeneity found in the meta-analyses of pharmacological treatments for mental health outcomes, with a median of the tau^2 distribution of 0.049 and an inter-quartile range of 0.010 to 0.242 (Rhodes 2015), and the heterogeneity was considered low when the estimated tau^2 was below the 25% quartile, moderate when between 25% and 50% of the quartile, and high when above the 50% quartile.

Huhn M, et al. Lancet 2019;394(10202):939-51

Rhodes KM, et al. J Clin Epidemiol 2015;68(1):52-60

**We evaluated local heterogeneity for ARI-F only. **Outcomes for which $I^2 \geq 50\%$ ($I^2 \geq 50\%$ considered to indicate considerable heterogeneity).

Table S2. Global and local heterogeneity and global inconsistency in the secondary meta-analysis

	tau^2	Design-by-treatment interaction model (p value)*
MADRS	0.000	0.989
Non-response rate	0.000	0.934
Non-remission rate	0.000	0.922
CGI-S	0.000	0.669
Social function	0.002	0.783
All-cause discontinuation	0.124	0.170
Discontinuation due to adverse events	0.000	0.574
At least one adverse event**	0.023	0.084
Serious adverse event	0.000	0.347
Akathisia**	0.522	0.044
Tremor**	0.775	0.034
Weight gain	0.000	0.625

*$p < 0.1$ was considered as considerable global inconsistency.

**We evaluated local heterogeneity for ARI-F only. **Outcomes for which $I^2 \geq 50\%$.

Table S3. Characteristics of the double-blind, randomized, placebo-controlled trials of brexpiprazole included in the subgroup pairwise meta-analysis

(1) Study, (2) country, (3) study duration and study design, (4) sponsorship	Patient inclusion criteria	Drug (dosage), n	Titration	MADRS at baseline	Mean age (mean±SD, y)/male (%)	Race (%)	Combined AD (dose, mg/d)
(1) Hobart 2018 (NCT02196506), (2) international, (3) 6 w, DBRPCT, (4) industry	Adult out-pt (age 18~65 y) with MDD (DSM-IV-TR) who had an inadequate response to 1~3 AD tx (MGHATRQ<50% improvement), current depressive episode≥8 w, HAMD17≥18	BRE 2 mg/d (fixed), 192	1st w: 0.5 mg/d<br>2nd w: 1.0 mg/d<br>3rd w: 2.0 mg/d	27.1±5.7	43.0±12.7/23.4	White 85.4	DUL40~60, ESC10~20, FLUO20~40, PAR-CR37.5~50, SER100~200, VEN-XR75~225
		PLA, 202		26.2±6.2	42.7±12.5/28.7	White 84.7	
(1) Kato 2023 (NCT03697603), (2) Japan, (3) 6 w, DBRPCT, (4) industry	Adult out-pt (age 20~64 y) with MDD (DSM-5) who had an inadequate response to 1~3 AD tx (<50% improvement on patient self-evaluation), current depressive episode≥8 w, HAMD 17≥18	BRE 2 mg/d (fixed), 246	1st w: 1 mg/d<br>2nd w: 2.0 mg/d	26.9±6.9	40.0±10.7/58.4	Japanese 100.0	DUL20~60, ESC10~20, FLUV50~150, MIL25~100, PAR-CR12.5~50, PAR-IR10~40, SER25~100, VEN-XR37.5~225
		BRE 1 mg/d (fixed), 250	No titration	26.7±6.4	40.9±10.8/53.2	Japanese 100.0	
		PLA, 244		27.3±6.2	39.8±10.8/56.8	Japanese 100.0	
(1) Thase 2015 (NCT01360632), (2) international, (3) 6 w, DBRPCT, (4) industry	Adult out-pt (age 18~65 y) with MDD (DSM-IV-TR) who had an inadequate response to 1~3 AD tx (MGHATRQ<50% improvement), current depressive episode≥8 w, HAMD 17≥18	BRE 3 mg/d (fixed), 230	1st w: 0.5 mg/d<br>2nd w: 1.0 mg/d<br>3rd w: 3.0 mg/d	26.4±5.2	44.5±11.2/32.2	Caucasian 87.4	DUL40~60, ESC10~20, FLUO20~40, PAR-CR37.5~50, SER100~200, VEN-XR75~225
		BRE 1 mg/d (fixed), 226	1st w: 0.5 mg/d<br>2nd w: 1.0 mg/d	26.7±5.6	45.7±11.6/30.1	Caucasian 81.0	
		PLA, 221		26.3±5.3	46.6±11.0/33.9	Caucasian 85.1	
(1) Thase 2015 (NCT01360645), (2) international, (3) 6 w, DBRPCT, (4) industry	Adult out-pt (age 18~65 y) with MDD (DSM-IV-TR) who had an inadequate response to 1~3 AD tx (MGHATRQ<50% improvement), current depressive episode≥8 w, HAMD 17≥18	BRE 2 mg/d (fixed), 188	1st w: 0.5 mg/d<br>2nd w: 1.0 mg/d<br>3rd w: 2.0 mg/d	26.6±5.8	44.1±11.6/30.9	White 86.7	DUL40~60, ESC10~20, FLUO20~40, PAR-CR37.5~50, SER100~200, VEN-XR75~225
		PLA, 191		27.1±5.6	45.2±11.3/28.3	White 86.9	

AD: antidepressant, ATRQ: Antidepressant Treatment Response Questionnaire, BRE: brexpiprazole, d: day, DBRPCT: Double-blind, randomized, placebo-controlled trial, DES: desvenlafaxine, DSM: Diagnostic and Statistical Manual of Mental Disorders, DUL: duloxetine, ESC: escitalopram, FDA: Food and Drug Administration, FLUO: fluoxetine, FLUV: fluvoxamine, HAMD17: 17-item Hamilton Depression Rating Scale of depression, MADRS: Montgomery Åsberg Depression Rating Scale, MDD: major depressive disorder, MGHATRQ: Massachusetts General Hospital Antidepressant Treatment History Questionnaire, MIL: milnacipran, n: number of patients, PAR-IR(CR): paroxetine-immediate release (controlled release), PLA: placebo, pt: patient, SD: standard deviation, SER: sertraline, tx: treatment, VEN-XR: venlafaxine-extended release, w: week, y: year

Appendix S1. PRISMA for Network Meta-Analyses Checklist.[1]

Section/Topic	Item #	Checklist Item	Reported on Page #
**TITLE**			1-
Title	1	Identify the report as a systematic review *incorporating a network meta-analysis (or related form of meta-analysis).*	
**ABSTRACT**			3-
Structured summary	2	Provide a structured summary including, as applicable:<br>**Background:** main objectives<br>**Methods:** data sources; study eligibility criteria, participants, and interventions; study appraisal; and *synthesis methods, such as network meta-analysis.*<br>**Results:** number of studies and participants identified; summary estimates with corresponding confidence/credible intervals; *treatment rankings may also be discussed. Authors may choose to summarize pairwise comparisons against a chosen treatment included in their analyses for brevity.*<br>**Discussion/Conclusions:** limitations; conclusions and implications of findings.<br>**Other:** primary source of funding; systematic review registration number with registry name.	
**INTRODUCTION**			4-
Rationale	3	Describe the rationale for the review in the context of what is already known*, including mention of why a network meta-analysis has been conducted.*	
Objectives	4	Provide an explicit statement of questions being addressed, with reference to participants, interventions, comparisons, outcomes, and study design (PICOS).	
**METHODS**			5-
Protocol and registration	5	Indicate whether a review protocol exists and if and where it can be accessed (e.g., Web address); and, if available, provide registration information, including registration number.	
Eligibility criteria	6	Specify study characteristics (e.g., PICOS, length of follow-up) and report characteristics (e.g., years considered, language, publication status) used as criteria for eligibility, giving rationale. *Clearly describe eligible treatments included in the treatment network, and note whether any have been clustered or merged into the same node (with justification).*	
Information sources	7	Describe all information sources (e.g., databases with dates of coverage, contact with study authors to identify additional studies) in the search and date last searched.	
Search	8	Present full electronic search strategy for at least one database, including any limits used, such that it could be repeated.	
Study selection	9	State the process for selecting studies (i.e., screening, eligibility, included in systematic review, and, if applicable, included in the meta-analysis).	
Data collection process	10	Describe method of data extraction from reports (e.g., piloted forms, independently, in duplicate) and any processes for obtaining and confirming data from investigators.	

Data items	11	List and define all variables for which data were sought (e.g., PICOS, funding sources) and any assumptions and simplifications made.	
**Geometry of the network**	**S1**	Describe methods used to explore the geometry of the treatment network under study and potential biases related to it. This should include how the evidence base has been graphically summarized for presentation, and what characteristics were compiled and used to describe the evidence base to readers.	
Risk of bias within individual studies	12	Describe methods used for assessing risk of bias of individual studies (including specification of whether this was done at the study or outcome level), and how this information is to be used in any data synthesis.	
Summary measures	13	State the principal summary measures (e.g., risk ratio, difference in means). *Also describe the use of additional summary measures assessed, such as treatment rankings and surface under the cumulative ranking curve (SUCRA)* values, as well as modified approaches used to present summary findings from meta-analyses.*	
Planned methods of analysis	14	Describe the methods of handling data and combining results of studies for each network meta-analysis. This should include, but not be limited to:<br>• *Handling of multi-arm trials;*<br>• *Selection of variance structure;*<br>• *Selection of prior distributions in Bayesian analyses; and*<br>• *Assessment of model fit.*	
**Assessment of Inconsistency**	**S2**	Describe the statistical methods used to evaluate the agreement of direct and indirect evidence in the treatment network(s) studied. Describe efforts taken to address its presence when found.	
Risk of bias across studies	15	Specify any assessment of risk of bias that may affect the cumulative evidence (e.g., publication bias, selective reporting within studies).	
Additional analyses	16	Describe methods of additional analyses if done, indicating which were pre-specified. This may include, but not be limited to, the following:<br>• Sensitivity or subgroup analyses;<br>• Meta-regression analyses;<br>• *Alternative formulations of the treatment network; and*<br>• *Use of alternative prior distributions for Bayesian analyses (if applicable).*	
**RESULTS†**			7-
Study selection	17	Give numbers of studies screened, assessed for eligibility, and included in the review, with reasons for exclusions at each stage, ideally with a flow diagram.	
**Presentation of network structure**	**S3**	Provide a network graph of the included studies to enable visualization of the geometry of the treatment network.	

**Summary of network geometry**	**S4**	Provide a brief overview of characteristics of the treatment network. This may include commentary on the abundance of trials and randomized patients for the different interventions and pairwise comparisons in the network, gaps of evidence in the treatment network, and potential biases reflected by the network structure.	
Study characteristics	18	For each study, present characteristics for which data were extracted (e.g., study size, PICOS, follow-up period) and provide the citations.	
Risk of bias within studies	19	Present data on risk of bias of each study and, if available, any outcome level assessment.	
Results of individual studies	20	For all outcomes considered (benefits or harms), present, for each study: 1) simple summary data for each intervention group, and 2) effect estimates and confidence intervals. *Modified approaches may be needed to deal with information from larger networks.*	
Synthesis of results	21	Present results of each meta-analysis done, including confidence/credible intervals. *In larger networks, authors may focus on comparisons versus a particular comparator (e.g. placebo or standard care), with full findings presented in an appendix. League tables and forest plots may be considered to summarize pairwise comparisons.* If additional summary measures were explored (such as treatment rankings), these should also be presented.	
**Exploration for inconsistency**	**S5**	Describe results from investigations of inconsistency. This may include such information as measures of model fit to compare consistency and inconsistency models, *P* values from statistical tests, or summary of inconsistency estimates from different parts of the treatment network.	
Risk of bias across studies	22	Present results of any assessment of risk of bias across studies for the evidence base being studied.	
Results of additional analyses	23	Give results of additional analyses, if done (e.g., sensitivity or subgroup analyses, meta-regression analyses, *alternative network geometries studied, alternative choice of prior distributions for Bayesian analyses,* and so forth).	
**DISCUSSION**			10-
Summary of evidence	24	Summarize the main findings, including the strength of evidence for each main outcome; consider their relevance to key groups (e.g., healthcare providers, users, and policy-makers).	
Limitations	25	Discuss limitations at study and outcome level (e.g., risk of bias), and at review level (e.g., incomplete retrieval of identified research, reporting bias). *Comment on the validity of the assumptions, such as transitivity and consistency. Comment on any concerns regarding network geometry (e.g., avoidance of certain comparisons).*	
Conclusions	26	Provide a general interpretation of the results in the context of other evidence, and implications for future research.	
**FUNDING**			
Funding	27	Describe sources of funding for the systematic review and other support (e.g., supply of data); role of funders for the systematic review. This should also include information regarding whether funding has been received from manufacturers of treatments in the network and/or whether some of the authors are content experts with professional conflicts of interest that could affect use of treatments in the network.	13

1. Hutton B, Salanti G, Caldwell DM et al. The PRISMA extension statement for reporting of systematic reviews incorporating network meta-analyses of health care interventions: checklist and explanations. Ann Intern Med 2015; 162: 777-84.
